# When Social Media Shapes Appearance Consciousness: Parental Mediation in Problematic Media Usage and Emotional Intelligence Profiles

**DOI:** 10.11621/pir.2025.0406

**Published:** 2025-12-01

**Authors:** Elvira de Armas Cortés, José Luis Martínez Torresa, Jorge Enrique Torralbas Osléa

**Affiliations:** a University of Havana, Cuba

**Keywords:** problematic social media Use, emotional intelligence, parental mediation, adolescence, body image, appearance consciousness, gender

## Abstract

**Background.:**

Problematic social media use and emotional regulation difficulties have been associated with increased appearance-related concerns among adolescents. However, these associations may vary depending on the interaction of psychosocial factors and contextual influences such as parental mediation.

**Objective.:**

To identify profiles based on adolescents’ levels of problematic social media use and online emotional intelligence, and to examine their relationship with appearance-related social media consciousness, considering the mediating role of parental mediation strategies and the moderating role of gender.

**Design.:**

A total of 636 Cuban adolescents aged 12 to 15 participated in the study, the majority of whom were female (n = 351, 55.2%). Measures included validated scales of social media disorder, online emotional intelligence, parental mediation, and appearance-related social media consciousness (ASMC). Latent Profile Analysis was used to identify profiles, followed by parallel multiple mediation and moderated mediation models using the PROCESS macro.

**Results.:**

Four latent profiles were identified, reflecting different combinations of emotional competence and problematic use. Only active parental mediation emerged as a significant mediator between profiles and appearance-related social media consciousness (ASMC), with stronger effects among adolescents with lower emotional intelligence. No significant moderated mediation by gender was found, although girls reported higher exposure to mediation and more pronounced indirect effects in high-risk profiles.

**Conclusion.:**

These findings emphasize that adolescents’ digital and emotional profiles shape how appearance-related risks develop in social media contexts. They underscore the protective value of active parental mediation and suggest that interventions should not only address problematic social media use but also promote emotional competencies and strengthen family involvement, offering practical guidelines for prevention programs and school-based interventions.

## Introduction

The intensive use of digital social networks during adolescence has been linked to various mental health risks, especially concerning the construction of self-image and the development of emotional self-regulation skills (Cai et al., 2023; Charmaraman et al., 2025; Fathurohman et al., 2023). These social networks are key environments for socialization, participation, and identity exploration (Pérez-Torres, 2024; Senekal et al., 2023); however, their algorithmic functioning and the culture of constant exposure may promote problematic dynamics such as compulsive use, the pursuit of immediate social validation, and the internalization of unrealistic beauty standards (Casale & Banchi, 2020; Marengo et al., 2022; van Oosten et al., 2023).

### Problematic Use of Social Media and Online Emotional Intelligence

Problematic use is characterized by a loss of control over connection time, interference in other areas of daily life, and a pattern of psychological dependence, with harmful effects on overall well-being (Boer et al., 2022). During adolescence, these vulnerabilities can be explained by the mismatch between the neurocognitive maturation of the limbic and prefrontal systems, which influences sensitivity to social rewards, emotional self-regulation, and decision-making in digital environments (Meredith & Silvers, 2024; Pérez de Albéniz et al., 2021). Consequently, practices such as sexting, overexposure of body image, passive consumption of aesthetic content, and fear of missing out (FOMO) are increasingly common and can negatively affect self-esteem and subjective well-being (Groenestein et al., 2024; Taylor & Armes, 2024).

Deficits in emotional intelligence have been documented to increase adolescents’ vulnerability to these risks (Sánchez-López et al., 2022; Soriano-Sánchez & Jiménez-Vázquez, 2023). Emotional intelligence is a skill that involves recognizing, understanding, and regulating one’s own and others’ emotions, and it has been associated with lower dysfunctional technology use (Haro Gavidia & Mayorga Lascano, 2024).

Previous studies associate problematic use of social networks with lower emotional regulation capacities at this age (Alshakhsi et al., 2022; Aranda et al., 2022; Chen et al., 2025; Flack et al., 2024; Piccerillo & Digennaro, 2025).

Few studies have explored other possible OR correlations between these variables. Identifying combined profiles can help to understand specific patterns of risk and protection, rather than assuming uniform efects as traditionally studied. Taking this into account, the first objective of this study is to identify distinguishable psychosocial proiles of adolescents based on their levels of problematic social network use and emotional intelligence. It is expected that natural groupings with distinct patterns of risk and emotional resources will emerge, leading to the formulation of

**Hypothesis H1:** There are distinguishable profiles of adolescents according to their levels of problematic social media use and online emotional intelligence.

### Appearance Consciousness on Social Media

Problematic social media use and low levels of emotional intelligence may increase appearance consciousness on social media. The “appearance consciousness” construct is deined as the extent to which individuals’ thoughts and behaviors relect constant attention to how they might be perceived in terms of physical attractiveness by their audience on these platforms (Choukas-Bradley et al., 2020). This consciousness is associated with intensive exposure to visual content and with compulsive usage patterns, which are exacerbated when adolescents present deficits in emotional regulation (Çinaroglu & Yilmazer, 2025; Sari et al., 2023; Sommerfeld & Dror, 2025).

This concern with maintaining an idealized image has been linked to the pursuit of external validation, social comparison, and potential distortions of body image, with particularly notable effects among adolescent girls (Gioia et al., 2020; Saiphoo & Vahedi, 2019). This process cannot be understood solely as an individual psychological phenomenon, but rather as the result of a complex network of social, cultural, and technological norms that regulate how bodies are perceived, exposed, and evaluated. Body image is deeply shaped by processes of normalization, performativity, and social regulation, especially among young women (Berry et al., 2021; Bordo, 2023; Butler, 2011; McRobbie, 2009; Sœle et al., 2021). From these critical perspectives, it can be understood as a contemporary form of internalized surveillance and emotional management of the body based on the perception of others (Lupton, 2016). In this sense, the present study draws on critical perspectives that conceptualize body image not only as a psychological phenomenon, but also as part of broader processes of normalization, performativity, and cultural regulation.

Based on these findings, the second objective of this study is to examine the relationship between the proiles identiied from the combination of problematic social media use and adolescents’ emotional intelligence, and the level of appearance consciousness on social media. Accordingly, the following hypothesis is proposed:

**Hypothesis H2:** Profiles characterized by higher problematic social media use and lower emotional intelligence are associated with higher levels of appearance consciousness on social media.

### Parental Mediation as a Protective Factor

Previous studies have shown that the strategies used by mothers and fathers to support their children’s digital media use directly influence their online habits (Vossen et al., 2024), and parental mediation acquires particular importance. A positive family relationship has been linked to a lower propensity for problematic social media use, as well as to a reduction in risk behaviors and addictive tendencies during adolescence (de Vries et al., 2019; Faltynková et al., 2020; Livingstone & Helsper, 2008; Lu-kavská et al, 2022; Tan et al., 2025). Strategies based on dialogue and the promotion of autonomy have proven to be more effective than those of an exclusively restrictive nature, as they encourage greater internalization of norms and more reflective use of digital technologies (Beyens et al., 2024).

A signiicant association has also been found between parenting styles and emotional intelligence. Authoritarian styles have been negatively associated with this capacity, while democratic styles tend to foster its development (Wang et al., 2019). Moreover, adolescents who express positive feelings toward their parental figures tend to exhibit a healthier self-image and self-concept (Hashimoto et al., 2011).

These findings suggest that parental mediation not only influences problematic social media use and adolescents’ emotional intelligence, but may also have an impact on how appearance consciousness is constructed in digital environments. From this perspective, it is relevant to consider the articulating role that parental practices may play in the interaction among these three aspects of adolescent development.

Parental mediation strategies are not applied uniformly; rather, they may differ depending on individual characteristics such as gender. It has been observed that mothers and fathers tend to apply differentiated styles, often reproducing gender stereotypes that can affect the degree of supervision and the type of guidance provided (Wang & Cheung, 2023).

From this standpoint, the third objective is to analyze the mediating role of parental mediation strategies in the relationship between the identified psychosocial profiles and appearance consciousness on social media, also considering the adolescent’s gender. Accordingly, the following hypotheses are proposed:

**H3:** Parental mediation strategies mediate the relationship between psychosocial proiles and appearance consciousness on social media.

**H4:** The indirect effects between psychosocial profiles and appearance consciousness, mediated by parental mediation, difer according to the adolescent’s gender.

## Method

### Participants

A convenience sampling method was used, based on a random selection of schools located in four municipalities of Havana, Cuba. The sample consisted of 636 students enrolled in lower secondary education (Secundaria Básica), with ages ranging from 12 to 15 years (M = 14.0; *SD* = .721). The majority of participants were female (n = 351, 55.2%). The selected areas were linked to regions with different municipal human development indicators: central area (very high), southwest (high), central-south (medium), and central-north (low), with proportions of approximately 26%, 23%, 30%, and 21% of the total sample, respectively.

Inclusion criteria were as follows: (1) being an adolescent between 12 and 15 years of age; (2) being enrolled in classrooms randomly selected within the participating schools; and (3) having provided informed consent along with parental or legal guardian consent.

The response rate was 97%. In the remaining 3%, students began the questionnaire but did not complete it; their data were therefore excluded from the analyses. Measures were taken to minimize social desirability bias, including assurance of anonymity, voluntary participation, and the absence of teachers during data collection.

### Measures

*Problematic social media use:* The 9-item Social Media Disorder Scale (SMD; Boer et al., 2022) was used. Responses were dichotomous (1 = yes; 0 = no), and the total score was calculated by summing individual item scores. The scale has been validated cross-nationally in adolescent populations; it has not yet been adapted to the Cuban context. Reliability in the present study was acceptable (α = .71).

*Emotional intelligence on the Internet:* The Internet Emotional Intelligence Scale (IEIS, González-Cabrera et al., 2016) was used. It consists of 15 items across three dimensions: online emotional attention (M = 1.98, *SD* = .96), online emotional clarity (M = 2.40, *SD* = 1.17), and online emotional regulation (M = 2.61, *SD* = 1.20). Responses were given on a 5-point Likert scale (1 = never to 5 = always). Acceptable internal consistency values were obtained for each subscale (α attention = .74; α clarity = 0.82; α regulation = .81) and for the total scale (α = .89). A single principal component was extracted from the correlations between the three dimensions of emotional intelligence using Varimax rotation, explaining 70.2% of the variance (BTS: χ^2^(3) = 573, p < .001; KMO = .683). The scale has been validated in adolescent populations but not adapted to the Cuban context.

*Parental mediation on social media:* The Parental Mediation Scale for Social Media (Ho et al., 2020) was used. The scale consists of 16 items, measuring four parental mediation strategies: active mediation (M = 4.09, *SD* = 1.96), restrictive mediation (M = 2.72, *SD* = 1.66), authoritarian monitoring (M = 2.64, *SD* = 1.70), and non-intrusive inspection (M = 3.53, *SD* = 1.96). Responses were given on a 7-point Likert scale (1 = never to 7 = always). Internal consistency was acceptable for all strategies (α active = .76; α restrictive = .83; α authoritarian = .82; α non-intrusive = .73) and for the total scale (α = .91). Th e scale has been validated in adolescent populations but not specifically in Cuba.

*Appearance-related social media consciousness:* The Appearance-Related Social Media Consciousness Scale (ASMC; Choukas-Bradley et al., 2020) was used. It includes 13 items with a 7-point Likert scale (1 = never to 7 = always). Internal consistency in the present study was acceptable (α = .76). The scale was validated in adolescents but not adapted to the Cuban context.

### Procedure

Data were collected between April and May 2024. The instruments were self-administered in the classroom during school hours, in the presence of the research team, who provided general instructions, answered questions during the process, and ensured conditions of conidentiality. Adolescents participated in the study voluntarily, with written informed consent obtained from their parents or legal guardians. Informed consent was obtained offline through the administration of the educational institutions, in accordance with ethical guidelines. The study received approval from the Ethics Committee of the Faculty of Psychology at Havana University, Cuba, and was conducted following ethical principles and the Declaration of Helsinki. Participants were provided with a brief description of the study and assured of anonymity and conidentiality.

Data analysis was conducted using Jamovi 2.3.38 and SPSS 25.0. First, to identify distinguishable psychosocial proiles based on problematic social media use and online emotional intelligence, a Latent Profile Analysis (LPA) was performed. Th e indicators included in the model were the total scores for problematic use and the principal component score extracted from the dimensions of emotional intelligence. The model fit was evaluated using the Akaike Information Criterion; the Bayesian Information Criterion; Classification Likelihood Criterion; Sample-size Adjusted and Entropy (Akaike, 1987; Celeux & Soromenho, 1996; Schwarz, 1978).

**Figure 1.**
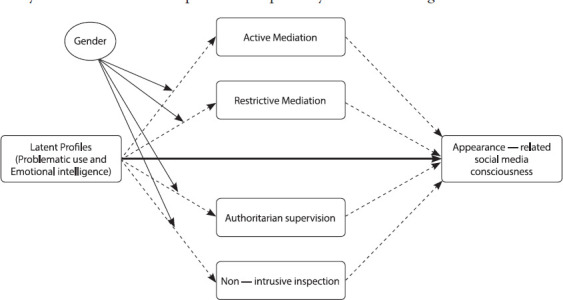
General conceptual model of the research

Subsequently, a parallel multiple mediation model and a moderated parallel multiple mediation model were conducted using PROCESS macro-Models 4 and 7 for SPSS, respectively (Hayes, 2022). These models allowed for: (1) estimating the behavior of appearance-related social media consciousness based on the latent proiles obtained; (2) exploring the mediating efects of parental mediation strategies in the relationship between the two aforementioned variables; and (3) examining the moderating effect of gender between the latent profiles and parental mediation strategies. The latent profiles were treated as a multicategorical independent variable; to include them as the antecedent variable in the model, a coding process was applied to create indicators (Hayes & Preacher, 2014). The latent profiles were ordered from those describing lower problematic social media use and higher emotional intelligence to those characterized by higher problematic use and lower emotional intelligence. Parental mediation strategies functioned as mediating variables, appearance-related social media consciousness as the dependent variable, and gender as the moderator. To estimate the specific relative indirect effects, a 95% confidence interval based on 5.000 bootstrap samples was used. If the conidence intervals did not include zero, the specific relative indirect effects were considered statistically significant. These analyses followed the conceptual model pathway described in *Figure 1*.

## Results

Participants were grouped according to their levels of problematic social media use and online emotional intelligence through Latent Profile Analysis. Models ranging from two to five classes were estimated (*Table 1*). The four-class solution was selected as the inal model for analysis based on its it indices, interpretability, and conceptual coherence. Fit indices considered included LogLik, AIC, BIC, CLC, SABIC, Entropy, posterior probabilities, and class sizes.

**Table 1:** Fit Indices of Latent Profiles Models

Profiles	LogLik	AIC	BIC	CLC	SABIC	Entropy
1	–2267	4542	4559	4536	4547	1.000
2	–2226	4466	4497	4453	4475	.605
3	–2226	4471	4516	4452	4484	.387
4	–2202	4430	4487	4405	4446	.629
5	–2195	4421	4492	4390	4442	.693

*Note: LogLik, Log Likelihood; AIC, Akaike Information Criterion; BIC, Bayesian Information Criterion; CLC, Classification Likelihood Criterion; SABIC, Sample-size Adjusted BIC*.

Although the ive-proile solution showed slightly better values for some it indices (LogLik, AIC, SABIC, ICL, entropy = .693), one class represented only 2.2% of the sample, raising concerns about stability and interpretability. In contrast, the four-proile solution presented slightly lower it indices (LogLik, AIC, SABIC, ICL, entropy = .629), but the BIC was lower than in the five-profile solution, all classes were adequately sized (minimum 10.2%), and posterior classification probabilities were satisfactory (min = .742, max = .845). Considering these metrics, conceptual interpretability, and consistency with prior literature, the four-proile solution was retained for further analysis.

**Figure 2.**
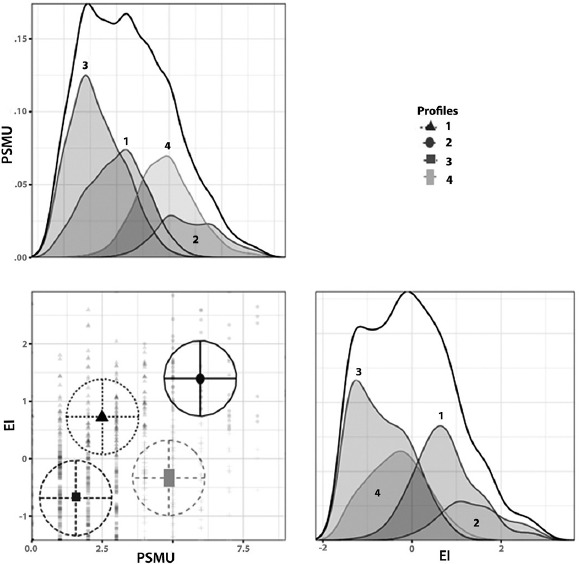
Densities and bivariate relationship between the variables estimated in the latent profiles analysis

*Notes: PSMU: Problematic social media use; EI: Emotional Intelligence*.

*Figure 2* shows the density plots and the bivariate relationship between the estimated variables for each proile. Based on this information, the latent proiles were characterized according to the levels of problematic social media use and online emotional intelligence of the adolescents belonging to each group.

Profile 1 (n = 170; 26.7%): Includes participants with high levels of emotional intelligence (M = .88, *SD* = .57) and low levels of problematic social media use (M = 2.45, *SD* = 1.17).

Profile 2 (n = 65; 1.2%): Characterized by the highest levels of both emotional intelligence (M = 1.47, SD = .62) and problematic social media use (M = 6.17, SD = 1.22).

Profile 3 (n = 248; 39.0%): Associated with the lowest levels of both emotional intelligence (M = -.74, SD = .54) and problematic social media use (M = 1.43, SD = 1.02).

Profile 4 (n = 153; 24.1%): Characterized by low emotional intelligence (M = -.40, SD = .56) and high levels of problematic social media use (M = 5.05, SD = 1.10).

The latent profiles were ordered from those describing lower levels of problematic social media use and higher emotional intelligence to those characterized by higher problematic use and lower emotional intelligence. Additionally, they were grouped into fictitious conditions through a process of indicator coding (*Table 2*); this allowed their inclusion as a multicategorical antecedent variable in the subsequent mediation and moderated mediation models, as well as the comparison of results based on levels of problematic social media use and online emotional intelligence. These findings provide an exploratory characterization of how adolescents combine emotional skills and social media engagement patterns. Results should be interpreted with caution due to the exploratory nature of the study and the entropy values observed.

**Table 2:** Dummy Conditions of Latent Profiles

Conditions	Description
C1	Profile 2 vs. Profile 1
C2	Profile 3 vs. Profile 1
C3	Proile 4 vs. Proile 1

**Figure 3.**
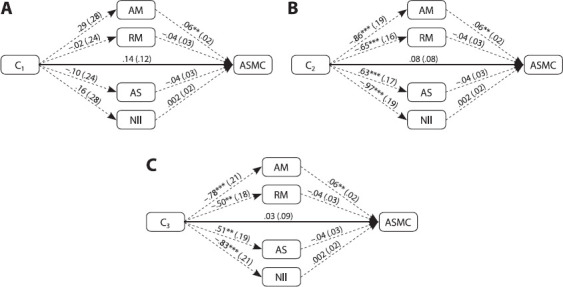
Estimated coefficients and standard errors of the parallel multiple mediation model

*Notes: An indicator coding process is used. C*_*1*_ *, Profile 2 vs. Profilel; C*_*2*_ *, Profile 3 vs. Profilel; C*_*3*_ , Profile 4 vs. Profile1. AM, Active mediation; RM, Restrictive mediation; AS, Authoritarian supervision; NII, Non-intrusive inspection. ASMC, Appearance-related social media consciousness. * p < .05; ** p <.01; ***p < .001

A parallel multiple mediation analysis was conducted to examine whether parental mediation strategies mediated the relationship between social media use proiles and appearance-related social media consciousness (*Figure 3*). As shown in *Table 3*, only active mediation signiicantly mediated the efects of proiles C_2_ and C_3_ compared to the reference profile C_1_. Other parental mediation strategies (restrictive mediation, authoritarian supervision, non-intrusive inspection) did not show signii-cant mediation efects.

**Table 3:** Relative Specific Effects of the Parallel Multiple Mediation Model

Mediation variables	Specific relative indirect effects in ASMC							
	AM		RM		AS		NII	
	Coeff (BootSE)	(BootLLCI, BootULCI)	Coeff (BootSE)	BootLLCI, BootULCI)	Coeff (BootSE)	(BootLLCI, BootULCI)	Coeff (BootSE)	(BootLLCI, BootULCI)
C1	.0166	(–.0128,	.0010	(–.0288,	–.0043	(–.0355,	.0004	(–.0165,
	(.0181)	.0585)	(.0133)	.0284)	(.0130)	.0207)	(.0075)	.0161)
C2	–.0493	(–.1006,	.0281	(–.0110,	–.0261	(–.0749,	–.0027	(–.0520,
	(.0234)	–.0108)	(.0221)	.0766)	(.0218)	.0118)	(.0239)	.0445)
C3	–.0449	(–.0940,	.0217	(–.0078,	–.0210	(–.0630,	–.0023	(–.0464,
	(.0222)	–.0089)	(.0189)	.0658)	(.0183)	.0092)	(.0208)	.0382)

*Notes: An indicator coding process is used. C1, Profile 2 vs. Profile1; C2, Profile 3 vs. Profile1; C3, Profile 4 vs. Profile1. AM, Active mediation; RM, Restrictive mediation; AS, Authoritarian supervision; NII, Non-intrusive inspection. Coeff., Coefficient; BootLLCI, lower level confidence interval; BootULCI, upper level confidence interval. 5.000 bootstrap samples were used*

**Figure 4.**
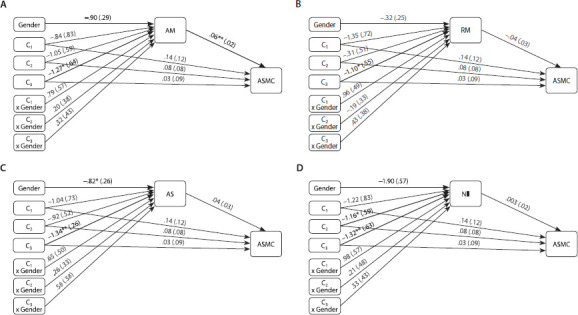
Coefficients and standard errors, moderated parallel multiple mediation model

Notes: An indicator coding process is used. CI, Profile 2 vs. Profilei; C2, Profile 3 vs. Profilei; C3, Profile 4 vs. Profilel. AM, Active mediation; RM, Restrictive mediation; AS, Authoritarian supervision; Nil, Non-intrusive inspection. ASMC, Appearance-related social media consciousness. Gender, 0= female, 1 =male. *p<.05; **p<.01; ***p<.001.

According to the estimates from the moderated parallel multiple mediation model (*Figure 4*), Condition C_2_ significantly predicted lower main effects on the non-intrusive inspection strategy compared to the reference profile, while Condition C_3_ predicted lower effects across all parental mediation strategies. The negative and signiicant efects of gender on the strategies of active mediation, authoritarian monitoring, and non-intrusive inspection indicate lower levels of parental mediation among male adolescents.

To explore the moderating effect of gender between the latent profiles and parental mediation, higher-order unconditional interaction tests were performed for each mediating variable. None of the tests confirmed a significant moderating effect of gender on the relationship between the independent variable and the mediators. Only for restrictive mediation were values close to signiicance for moderation obtained (R2 = .0116, F (3.628) = 2.5476, *p* = .055). Due to the lack of statistically significant interaction effect, the conditional effects of the profiles at different levels of the moderator (gender) are not interpreted.

The test for moderated mediation was conducted by inspecting the bootstrapped conidence intervals for the Index of Moderated Mediation for each proile contrast and each mediator. As shown in *Table 4*, none of these indices were statistically significant, as all confidence intervals contained zero. This provides no empirical support for the hypothesis that gender moderates any of the indirect pathways from the latent profiles to appearance-related social media consciousness.

**Table 4:** Index of Moderated Mediation, Moderated Parallel Multiple Mediation Model

Mediation variables	Specific relative indirect effects in ASMC							
	AM		RM		AS		NII	
	Coeff (BootSE)	(BootLLCI, BootULCI)	Coeff (BootSE)	BootLLCI, BootULCI)	Coeff (BootSE)	(BootLLCI, BootULCI)	Coeff (BootSE)	(BootLLCI, BootULCI)
C1	.0458	(–.0175,	–.0418	(–.1361,	.0270	(–.0210,	.0027	(–.0507,
	(.0409)	.1415)	(.0396)	.0193)	(.0339)	.1128)	(.0267)	.0624)
C2	.0120	(–.0341,	.0082	(–.0248,	.0107	(–.0223,	–.0006	(–.0189,
	(.0246)	.0659)	(.0189)	.0557)	(.0190)	.0569)	(.0101)	.0245)
C3	.0187	(–.0280,	–.0185	(–.0766,	.0239	(–.0134,	.0009	(–.0242,
	(.0279)	.0840)	(.0240)	.0182)	(.0255)	.0870)	(.0129)	.0316)

*Notes: An indicator coding process is used. C1, Profile 2 vs. Profilei; C2, Profile 3 vs. Profilei; C3, Profile 4 vs. Profilei. AM, Active mediation; RM, Restrictive mediation; AS, Authoritarian supervision; NII, Non-intrusive inspection. ASMC, Appearance-related social media consciousness. MMI, Moderated mediation index. BootLLCI, lower level confidence interval; BootULCI, upper level confidence interval. 5,000 bootstrap samples were used*

## Discussion

The present study aimed to identify differentiated adolescent profiles based on problematic social media use and online emotional intelligence, as well as to explore the relationship of these profiles with appearance-related social media consciousness and the mediating role of parental mediation strategies. The proposed hypotheses are discussed below.

*H1: There are distinguishable profiles of adolescents according to their levels of problematic social media use and online emotional intelligence*.

The results of the latent profile analysis support this hypothesis, identifying four distinct proiles of adolescents based on their levels of problematic social media use and online emotional intelligence. These profiles should not be interpreted as definitive or mutually exclusive categories, but rather as exploratory statistical groupings that capture heterogeneous patterns of adjustment in digital contexts. Their interpretative value lies less in providing closed typologies and more in opening hypotheses about the coexistence of risk and protective factors within adolescent populations.

The link between problematic social media and online emotional intelligence is based on complex conigurations of risk and protection, rather than a linear and uniform relationship.

The link between these categories is based on complex configurations of risk and protection, rather than a linear and uniform relationship between problematic social media use and emotional intelligence. Some patterns found align with those traditionally documented in the literature (Aranda et al., 2022; Beranuy et al., 2009; Chen et al., 2025; Parker et al., 2008; Piccerillo & Digennaro, 2025), which describe an inverse relationship between the two variables: high emotional intelligence and low problematic social media use (Proile 1), and low emotional intelligence and high problematic social media use (Proile 4). On the other hand, proiles were also found that relect less traditional patterns and a direct relationship between both variables: high emotional intelligence and problematic social media use (Proile 2), and low emotional intelligence and problematic social media use (Profile 3). Each of these conigurations carries important theoretical and practical implications.

The profiles that reflect an inverse relationship between problematic social media use and emotional intelligence represent the two ends of a continuum of costs and benefits of online interaction. *Profile 1*, composed of adolescents with high emotional intelligence and low problematic social media use, can be considered a digitally well-adjusted profile. For this group, the adaptive management of their digital socialization may allow them to beneit from the opportunities ofered by digital platforms while avoiding potential associated risks. In contrast, Proile 4, with low emotional intelligence and high problematic social media use, represents a particularly vulnerable coniguration, aligned with emotional dysregulation models that predict increased exposure to impulsive behaviors, compulsive gratiication-seeking, and harmful online content (Boer et al., 2022). The sharp contrast between these two profiles reinforces the idea that it is not the use of social media per se that is problematic, but rather the type of emotional and behavioral relationship adolescents develop with these platforms (Sala et al., 2024).

A proile characterized by high levels of emotional intelligence and high problematic social media use (*Proile 2)* challenges the traditional assumption that emotional intelligence always acts as a buffer against dysfunctional online behavior. This pattern may indicate that adolescents with well-developed emotional competencies do not always perceive problematic behaviors on social media. Therefore, it becomes necessary at this stage of life to ensure the presence of other protective factors for well-being in the face of potential risks — such as parental mediation.

The identification of a profile with low problematic social media use and low emotional intelligence (*Profile 3)* introduces a relevant interpretative possibility. Unlike the traditionally documented patterns, this proile does not suggest a harmonious functioning between the analyzed variables. It is possible that these adolescents are less involved in digital socialization — either by personal choice, external regulation, or lack of access — which could function as a form of passive protection against social media-related risks. However, this low level of involvement does not appear to translate into greater development of emotional competencies, which may indicate limited exposure to socially demanding interactions, both within and outside digital environments. Alternatively, it may also reflect broader emotional difficulties not necessarily linked to social media use, but rather to more general socialization processes. This lack of clear correspondence reinforces the idea that certain adolescent proiles do not follow linear patterns of risk or adjustment and must be understood from a contextual and exploratory perspective.

Our fi ndings underscore the value of profile analysis as a methodological approach, as it allows for the observation of latent interactions between variables that do not emerge as clearly in variable-centered approaches. The combination of online emotional intelligence and problematic use, when analyzed from a conigurational perspective, thus provides a more nuanced and ecological view of adolescent adjustment in digital environments.

Each of these configurations carries important theoretical and practical implications, but should be viewed as tentative insights that future research must test and reine across diferent cultural and developmental contexts.

*H2. Profiles characterized by higher problematic social media use and lower emotional intelligence are associated with higher levels of appearance consciousness on social media*.

In our study, proiles characterized by higher problematic social media use and lower emotional intelligence did not show signiicantly higher levels of appearance-related social media consciousness compared to proiles with healthy digital adjustment. Rather than acting solely as a protective factor, emotional intelligence may in these cases function as an adaptive resource that facilitates the emotional management of aesthetic exposure, without necessarily reducing the internalization of appearance norms (Verduyn et al., 2020). In line with critical perspectives, adolescents’ experiences in social media are deeply entangled with processes of cultural disciplining of the body and social regulation of femininity (Bordo, 2023; McRobbie, 2009). Within this framework, problematic use cannot be dissociated from the performative and normative dynamics that govern online visibility, where emotional intelligence may act both as a resource for managing exposure and as a mechanism reinforcing self-surveillance (Berry et al., 2021; Butler, 2011; Lupton, 2016; Singpliam, 2022). Taken together, these indings invite a reconsideration of traditional models of risk and protection in digital contexts, highlighting that adolescent adjustment must be understood not only in psychological but also in sociocultural and performative terms.

*H3: Parental mediation strategies mediate the relationship between psychosocial profiles and appearance-related social media consciousness*.

Among the parental mediation strategies evaluated, only active mediation emerged as a full mediator between the combined proiles of problematic social media use and emotional intelligence and appearance-related social media consciousness. In other words, the link between these psychosocial proiles and appearance-related concerns on social media, was not direct, but operated entirely through active parental mediation. Among the parental mediation strategies evaluated, only active mediation functioned as a signiicant mediating variable between the combined proiles of problematic social media use and emotional intelligence and appearance-related social media consciousness. This finding reinforces previous research highlighting the unique value of active mediation during adolescence, as it facilitates the development of critical skills when engaging with digital content (Chang et al., 2019; Liu et al.; 2023; Lwin et al., 2008).

However, our results also suggest that the efect of this mediating role is not homogeneous; rather, it operates more strongly in more vulnerable profiles, particularly among adolescents with lower emotional intelligence and more problematic social media exposure. This type of mediation appears to function as a compensatory resource which, through dialogical interaction with adults, enables processes of emotional regulation, critical reinterpretation of aesthetic standards, and symbolic construction of the digital body.

Conversely, adolescents with high levels of emotional intelligence may possess more autonomous tools for processing digital information, reducing the impact of parental mediation (Pluess & Belsky, 2013). In other words, the effectiveness of active mediation is not uniform; its modulatory value depends on the adolescent’s individual capacity to self-regulate their digital experience, proving more necessary and efective in those with greater emotional deicits or more limited coping skills.

By contrast, adolescents with high levels of emotional intelligence may already possess more autonomous tools for processing digital information, which reduces the relative impact of parental mediation (Pluess & Belsky, 2013). Thus, while active mediation fully explains the relationship under study, its efectiveness depends on the adolescent’s individual capacity to self-regulate their digital experience — proving more necessary and transformative in those with greater emotional deficits or limited coping skills.

In contrast, the absence of mediating effects from strategies such as authoritarian monitoring or restrictive mediation may indicate that, far from being neutral, these forms of intervention can be ineffective or even counterproductive. From a sociocul-tural perspective, the indings invite us to conceive parental mediation not merely as behavioral control, but as symbolic and cultural mediation, in which the adult acts as a meaning-making guide in response to the complexities of the digital ecosystem. Within this framework, it is the quality of parental support — rather than its intensity — that determines its transformative effectiveness.

*H4: The indirect relationship between psychosocial profiles and appearance-related social media consciousness, mediated by parental mediation, differ based on adolescent gender*.

The results did not provide evidence for a moderating effect of gender on the indirect relationship between psychosocial proiles and appearance-related social media consciousness, mediated by parental mediation. None of the indices of moderated mediation reached statistical signiicance, as all bootstrapped conidence intervals included zero. This lack of significance contrasts with previous studies documenting gender-based differences in the type and frequency of parental mediation strategies (Nikken & de Graaf, 2013; Wang & Cheung, 2023).

One possible explanation is that social media has increasingly aestheticized the male body, a process that was historically more restricted to females, thereby reducing traditional gender gaps in exposure to appearance-related pressures. New generations may be experiencing more egalitarian forms of digital engagement, in which both boys and girls face comparable demands of aesthetic self-presentation, external validation, and digital self-management.

Although non-significant, some conditional estimates suggest patterns that may be theoretically relevant. Active mediation appeared more influential among girls under conditions of high digital vulnerability, possibly reflecting heightened exposure to restrictive aesthetic norms and a greater need for parental support. By contrast, boys reported lower levels of exposure to all parental mediation strategies, which may limit the moderating role of the family environment in their digital experience. These trends, however, should be treated as exploratory rather than conclusive.

From a broader perspective, the indings suggest that gender, as operationalized in this study, may not fully capture the complexity of adolescent trajectories in digital contexts. Future research should consider intersectional or culturally situated approaches to gender and examine other potential moderators, such as sexual orientation, social class, or speciic parenting styles.

## Conclusions

This study provides empirical evidence on the existence of differentiated psychosocial proiles among adolescents based on their problematic social media use and online emotional intelligence, and their relationship with appearance-related awareness in digital environments. These profiles should be interpreted as exploratory rather than definitive, offering a heuristic framework to understand the complexity of adolescent trajectories in digital contexts.

The results also underscore that active mediation can serve as a key protective resource, especially among adolescents with lower emotional competence, reinforcing the crucial role of families in digital guidance. Although no signiicant gender moderation effects were observed, some non-significant tendencies suggested potential differential patterns worth exploring in future research.

Overall, these findings emphasize the importance of differentiated preventive and intervention strategies that take into account not only the level of social media use or individual vulnerability, but also the relational and emotional contexts in which adolescent digital experiences unfold.

The findings of this study offer relevant implications for psychoeducational interventions, family counseling, and the design of educational policies in digital environments.

First, the identification of differentiated adolescent profiles based on problematic social media use and emotional intelligence suggests that interventions should not be homogeneous, but rather tailored to the speciic risk conigurations and emotional resources of each group. Particular attention should be given to the profile characterized by high emotional intelligence (EI) and high problematic social media use, which challenges the traditional notion of EI as a protective factor. In these cases, it is advisable to foster critical emotional intelligence — one that not only helps regulate emotions but also enables questioning the aesthetic standards circulating on social media.

Second, the evidence of the mediating role of active parental mediation highlights the need to strengthen parental competencies for relective digital guidance. Workshops or training programs aimed at parents can promote dialogue, co-navigation, and the critical re-signification of digital content. Th ese programs should incorporate gender-sensitive strategies, recognizing, for instance, the lower receptiveness and lower exposure to mediation among adolescent boys, and providing families with targeted resources in this regard.

At the school and community level, the results support the design of integrated interventions that combine emotional intelligence development with critical digital literacy. Speciically, it is recommended to address content related to body image, aesthetic pressure, and beauty ideals on social media, all from a culturally situated and non-normative perspective. These actions could be part of cross-cutting school programs in digital education with a focus on gender and equity.

## Limitations

Among the study’s limitations, the exclusive use of self-reports stands out, which may introduce social desirability bias. Future research would beneit from complementing these measurements with parental reports, direct observations, or qualitative methodologies. Additionally, the study focused on adolescents from urban areas of Havana, which limits the generalizability of the findings. This study used a cross-sectional design, preventing causal inferences between problematic social media use, emotional intelligence, parental mediation, and appearance-related consciousness. Furthermore, other potentially relevant variables were not controlled for, including total time spent on social media and socio-economic status, which may have inluenced the observed associations. Comparative studies in rural contexts or other countries could enrich the understanding of the identified profiles.
